# Multi‐omics identifies microbiota‐derived deoxycholic acid as a key mediator of blood‐brain barrier dysfunction in Parkinson's disease

**DOI:** 10.1002/imt2.70076

**Published:** 2025-09-17

**Authors:** Zhe Zhao, Jing Chen, Yixuan Liu, Shiqi Wang, Danhua Zhao, Chaobo Bai, Meifang Wu, Gaofei Hu, Yiwen Fu, Lu Fang, Xiaoyi Liu, Zheng Zhang, Rui Zhan, Lemin Zheng, Junliang Yuan

**Affiliations:** ^1^ Department of Pharmacy Peking University Third Hospital Beijing China; ^2^ Institute for Drug Evaluation Peking University Health Science Center Beijing China; ^3^ Department of Neurology, Peking University Sixth Hospital, Peking University Institute of Mental Health, NHC Key Laboratory of Mental Health (Peking University) National Clinical Research Center for Mental Disorders (Peking University Sixth Hospital) Beijing China; ^4^ Neuroscience Research Institute and Department of Neurobiology, School of Basic Medical Sciences Peking University Beijing China; ^5^ Key Laboratory for Neuroscience, Ministry of Education/National Health Commission Peking University Beijing China; ^6^ Department of Cardiovascular Affiliated Hospital of Putian University Putian China; ^7^ The Institute of Cardiovascular Sciences and Institute of Systems Biomedicine, School of Basic Medical Sciences, State Key Laboratory of Vascular Homeostasis and Remodeling, NHC Key Laboratory of Cardiovascular Molecular Biology and Regulatory Peptides, Beijing Key Laboratory of Cardiovascular Receptors Research, Health Science Center Peking University Beijing China; ^8^ Research Center for Cardiopulmonary Rehabilitation, University of Health and Rehabilitation Sciences Qingdao Hospital (Qingdao Municipal Hospital), School of Health and Life Sciences University of Health and Rehabilitation Sciences Qingdao China; ^9^ Beijing Tiantan Hospital, China National Clinical Research Center for Neurological Diseases, Advanced Innovation Center for Human Brain Protection Capital Medical University 6 Tiantan Xili, Chongwen District Beijing China

## Abstract

The gut microbiota derived from Parkinson's disease patients, enriched in *Bacteroides fragilis* (*B. fragilis*) and *Phocaeicola vulgatus* (*P. vulgatus*), may promote the elevations of deoxycholic acid, iso‐deoxycholic acid, and ursodeoxycholic acid levels in the systemic circulation. The increased serum bile acids, in turn, contribute to the endothelial cell death and pericyte injury possibly through activating interferon alpha response and TGF‐β signaling pathways at the blood‐brain barrier in the midbrain, ultimately leading to the neurodegeneration and motor deficits in the germ‐free mice.

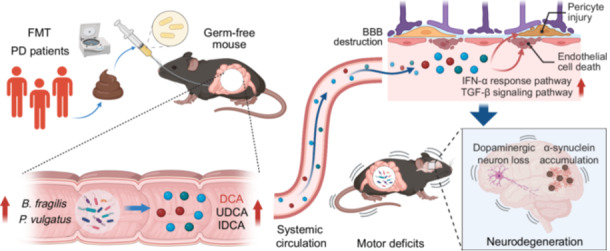

To the Editor,

Parkinson's disease (PD) is a common neurodegenerative disorder characterized by motor symptoms, associated with dopaminergic neuronal loss and α‐synuclein (α‐syn) accumulation in the substantia nigra (SN) [1]. Nevertheless, gastrointestinal (GI) dysfunction often precedes motor symptoms in patients, suggesting that PD might initiate from gut [2]. Consistently, accumulating evidence highlights the critical role of microbiota–gut–brain axis in PD [3]. Recent studies demonstrate that gut microbiota transplantation from PD patients into transgenic *α‐syn* overexpressing or A53T mice exacerbates motor deficits, GI dysfunction, and dopaminergic neuronal loss [4, 5]. However, the direct effects of PD patient‐derived fecal microbiota remain poorly defined.

In this study, we transplanted fecal microbiota from PD patients into germ‐free (GF) mice to assess whether it induces PD‐like phenotypes and to explore the underlying mechanisms. By integrating midbrain single‐nucleus RNA sequencing (snRNA‐seq), fecal metagenomics, and serum metabolomics, we explored how microbiota remodels midbrain cellular landscapes and identified which microbial metabolites mediate the gut‐brain interactions in PD.

### Gut microbiota transplantation from PD patients promotes PD phenotypes in the GF mice

Fecal samples were collected from five treatment‐naïve PD patients and five matched healthy controls (HCs). A total of 12 GF mice were randomly divided into two groups: the HC group mice receiving pooled fecal suspension from HCs, and the PD group mice receiving pooled fecal microbiota from PD patients (Figure 1A). The PD group mice exhibited significant weight loss (Figure S1A) and motor deficits, as measured by the open field test (OFT), rotarod test, and pole test (Figure 1B–D, Figure S1B–H). Additionally, GI function was remarkably impaired in the PD group mice, with reduced pellets, shorter colon lengths, and decreased fecal water concentrations (Figure 1E,F, Figure S1I). Moreover, immunohistochemical (IHC) analysis demonstrated that PD patient‐derived microbiota significantly decreased the number of tyrosine hydroxylase‐positive (TH^+^) neurons in the SN to approximately one‐third of that in the HC group (*p* < 0.01, Figure 1G,H). In addition, the optical density of α‐syn in the SN of the PD group mice was nearly twice that of the HC group mice (*p* < 0.01, Figure S1J,K). Collectively, these data demonstrate that gut microbiota from PD patients recapitulates clinical and pathological PD hallmarks in the GF mice.

**FIGURE 1:**
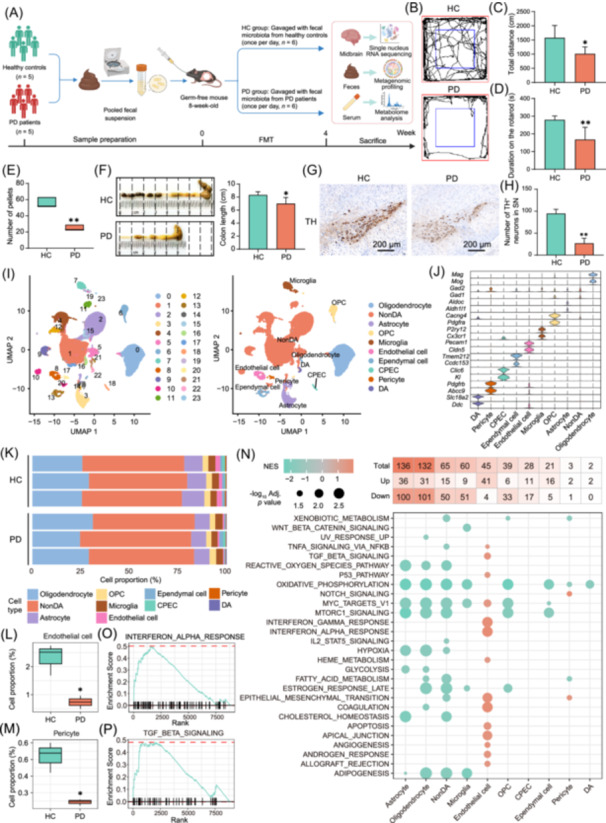
Gut microbiota transplantation from Parkinson's disease (PD) patients promotes PD phenotypes in the germ‐free (GF) mice. (A) Flow chart of experimental design. Created with BioRender.com. (B) Representative trajectory map of open field test. (C) Total distance traveled in the whole open field. (D) Rotarod test. (E) Number of total pellets. (F) Representative images of colon and colon lengths. (G) Representative images of immunohistochemical (IHC) staining of tyrosine hydroxylase‐positive (TH^+^) neurons in the SN. (H) Numbers of TH^+^ neurons in the substantia nigra (SN). (I) Uniform manifold approximation and projection (UMAP) illustration of cells colored by clusters (left) and cell types (right). (J) Violin plots of expression values of cell‐type‐specific markers for the cell types in the midbrain. (K) Proportions of different cell types in the midbrain. Cell type proportions of endothelial cells (L) and pericytes (M) in the midbrain. (N) The number of cell‐type‐specific differentially expressed genes (DEGs) and gene set enrichment analysis (GSEA) analysis of hallmark pathways in different cell types. Enrichment profiles of the interferon alpha response (O) and TGF‐β signaling (P) gene sets in endothelial cells. For the bar plot, the data are presented as mean ± standard deviation (SD). For the box plots: centerline, median; box, Interquartile Range (IQR, the range between the 25th and 75th percentiles); whiskers, 1.5 × IQR. For C–E, *n* = 6 in each group. For F, *n* = 5 in each group. For H–P, *n* = 3 in each group. Statistics calculated by Student's *t*‐test (C–D, F, and H), Mann–Whitney *U* tests (E and L, M), or GSEA analysis (N). In (C–H) and (L, M), *p < 0.05, **p < 0.01 versus the HC group. CPEC, choroid plexus epithelial cells; DA, dopaminergic; HC, healthy control; NES, normalized enrichment score; NonDA, non‐dopaminergic; OPC, oligodendrocyte precursor cells; PD, Parkinson's disease; SN, substantia nigra; TH, tyrosine hydroxylase; UMAP, Uniform manifold approximation and projection.

### Gut microbiota transplantation from PD patients remodels midbrain single‐cell transcriptomes

To further explore how PD patient‐derived microbiota drives neurodegeneration, we conducted snRNA‐seq on midbrain tissues. We captured single‐nucleus transcriptomes from an average of approximately 10,047 cells per sample, detecting an average of 2513 genes and 37,099 reads per cell. After quality control, 8542, 11,636, 10,667, 9648, 9250, and 10,341 qualified nuclei from each sample were retained for further analysis. Uniform Manifold Approximation and Projection analysis identified 24 distinct clusters (Figure 1I, Figure S2A) and 10 cell populations, including oligodendrocytes, microglia, oligodendrocyte precursor cells, non‐dopaminergic neurons, astrocytes, endothelial cells, ependymal cells, pericytes, choroid plexus epithelial cells, and dopaminergic (DA) neurons (Figure 1I). Cellular identities were confirmed through enrichment of cell type‐specific markers (Figure 1J). Notably, PD mice showed substantial shifts in cell type composition (Figures S2B, Figure 1K). Specifically, the key cellular components of the blood‐brain barrier (BBB), endothelial cells and pericytes, were reduced in the PD group mice (both *p* < 0.05, Figure 1L,M). Moreover, 2–136 differentially expressed genes were identified across cell types. Furthermore, gene set enrichment analysis of hallmark pathways highlighted the significant dysregulation in several immune‐related pathways (Figure 1N). Notably, interferon alpha response and TGF‐β signaling pathways were enriched in endothelial cells (Figure 1O,P). Besides, REACTOME analysis indicated the metabolic disturbances across cell types, like Selenoamino acid metabolism (Figure S2C). Intriguingly, Gene Ontology Biological Process (GO BP) enrichment highlighted the Neuron death and Neuron apoptotic process pathways in DA neurons and microglia (Figure S2D). Furthermore, we compared the expression of candidate genes with PD risk via altered expression identified by Kia DA et al. [6]. In the whole midbrain, *Apbb2*, *Mapt*, and *Sh3gl2* were downregulated and *Trpm7*, *Spats2l*, and *Ano3* were increased in the PD group (Figure S2E). Additionally, genes like *Mapt*, *Ano3*, and *Elovl7* were differentially expressed across different cell types (Figure S2F). These results together suggest that PD patient‐derived microbiota influences the expression of PD risk genes and cellular responses across different cell types in the midbrain of GF mice, potentially promoting PD development.

### Gut microbiota transplantation from PD patients alters gut microbiota community and serum metabolic profiles

Fecal metagenomic and serum metabolome analyses revealed the significant differences between the two groups. Alpha‐diversity analysis showed significantly elevated microbial diversity in the PD group (Figure 2A,B). Additionally, Anosim and principal coordinates analysis confirmed the distinct microbial structures (Figure 2C,D). Taxonomic profiling identified PD‐associated microbial shifts, including *Duncanella dubosii* depletion, enrichment of *Bacteroides fragilis (B. fragilis)* and *Phocaeicola vulgatus* (*P. vulgatus*) in the PD group mice (all *p* < 0.01; Figure 2E–I). Orthogonal partial least squares discriminant analysis (OPLS‐DA) of serum metabolites highlighted the significant group separation (Figure 2J). Furthermore, the differential analysis identified remarkably elevated iso‐deoxycholic acid (IDCA), deoxycholic acid (DCA), ursodeoxycholic acid (UDCA), indole‐3‐lactic acid, and hippuric acid in the PD group mice (all *p* < 0.01; Figure 2K,L). Overall, these findings suggest that gut microbiota may contribute to PD progression via modulating serum metabolite profiles.

**FIGURE 2:**
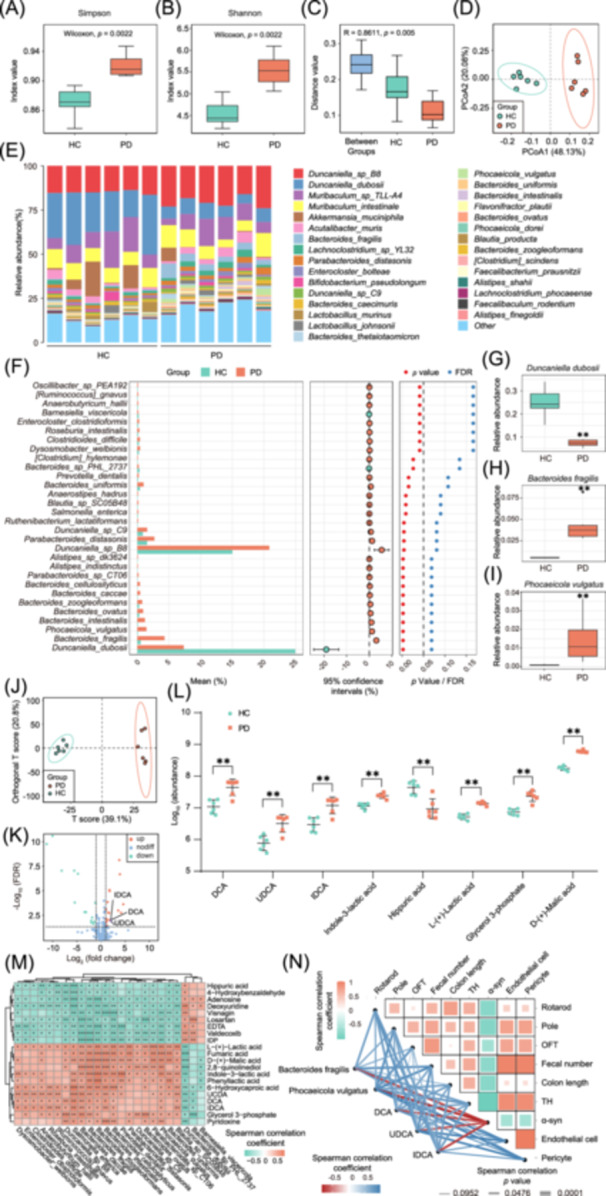
Gut microbiota transplantation from PD patients alters the gut microbiota community and serum metabolic profiles in the GF mice. Alpha‐diversity analysis indicated by Simpson (A) and Shannon (B). (C) Beta diversity based on Anosim method. (D) Principal coordinates analysis (PCoA) plots of beta diversity. (E) Relative abundances of gut microbiota at the species level in the different groups. (F) Statistical analysis of metagenomic profiles (STAMP) analysis of differentially abundant microbial species. Relative abundances of *Duncanella dubosii* (G), *Bacteroides fragilis* (H), and *Phocaeicola vulgatus* (I) in different groups. (J) Orthogonal partial least squares discriminant analysis (OPLS‐DA) plot of serum metabolomic profiles. (K) Volcano plot of differentially abundant metabolites in the serum from the PD group mice compared to the HC group mice. (L) Quantification of significantly differential metabolites in the serum. (M) Heatmap showing the association between differentially abundant serum metabolites and fecal microbial species. (N) The association between the key serum metabolites, microbial species, and experimental results. For the box plots: centerline, median; box, IQR (the range between the 25th and 75th percentiles); whiskers, 1.5 × IQR. For L, data are presented as medians with IQR. In this figure, *n* = 6 in each group. Statistics calculated by Mann–Whitney *U* tests (A–L) or Spearman correlation tests (M, N). **p < 0.01 versus the HC group. In (M, N), *p < 0.05, **p < 0.01, ***p < 0.001 for Spearman correlation. α‐syn, α‐synuclein; DCA, deoxycholic acid; FDR, false discovery rate; IDCA, iso‐deoxycholic acid; OFT, open field test; PCoA, principal coordinates analysis; PD, Parkinson's disease; TH, tyrosine hydroxylase; UDCA, ursodeoxycholic acid.

To further elucidate the molecular mechanisms underlying the PD‐like symptoms induced by patient‐derived microbiota, several correlation analyses were performed to integrate the multi‐omics data. First, the heatmap showed that *B. fragilis* and *P. vulgatus* abundances were positively correlated with the serum IDCA, DCA, and UDCA levels (Figure 2M). Furthermore, association analyses demonstrated the remarkable correlations among behavioral performance, GI function, dopaminergic loss, α‐syn accumulation, and midbrain compositions of endothelial cells and pericytes, which were also associated with the levels of microbial species and serum bile acids (Figure 2N). Collectively, these associations support that PD patient‐derived microbiota may elevate circulating bile acids, which impair the BBB integrity and drive neurodegeneration (Figure S3).

To validate the role of bile acids in PD progression, rotenone‐induced PD mice were fed with DCA for 4 weeks (Figure S4A). Bile acid quantification validated the increased DCA levels in the midbrains after DCA administration (Figure S4B). Compared to the Rotenone group mice, DCA treatment aggravated motor impairments and GI dysfunction (Figure S4C–I). Pathologically, DCA administration accelerated dopaminergic neurodegeneration in the SN (*p* < 0.05, Figure S4J,K). Moreover, transmission electron microscopy analysis showed disorganized tight junctions and damaged endothelial cells in the BBB of the Rotenone group mice. Meanwhile, DCA treatment further worsened the BBB impairment induced by rotenone (Figure S4L). These results together demonstrate that DCA treatment exacerbates PD‐like phenotypes and BBB impairment.

PD is the second most common neurodegenerative disorder, marked by motor and GI symptoms [1]. Gut microbiota dysbiosis is closely involved in PD pathogenesis [2]. However, the causal relationship remains unclear. To further explore this, we transplanted GF mice with gut microbiota from PD patients or HC donors. GF mice, lacking resident microbiota, eliminate confounders inherent in conventional mice and serve as the gold standard for studying microbial causality in neurological diseases [7]. Our study shows that PD patient‐derived microbiota transplantation recapitulates PD‐like phenotypes in GF mice, highlighting the potential causal role of gut microbiota in PD pathogenesis.

Recent studies have identified altered gut microbiota in PD patients and animals [4, 8, 9]. Our previous meta‐analysis showed depleted genera producing short‐chain fatty acids (SCFAs) and increased *Akkermansia* and *Bilophila* in PD patients [9]. To determine the critical microbes for PD, we performed fecal metagenomics in the GF mice. The alpha and beta diversity results revealed significant gut microbiota alterations in the PD group mice. At the species level, *B. fragilis* and *P. vulgatus* were enriched in the mice receiving PD microbiota. Notably, *B. fragilis*, from the *Bacteroides* genera, increases in the feces of PD patients [10]. Moreover, *Bacteroides uniform* was reported to regulate neuronal dopamine levels, indicating the potential effects of *Bacteroides* on PD [11]. Besides, *P. vulgatus* belongs to *Phocaeicola* genus, which exhibits associations with cognition in neurodevelopment [12]. Collectively, these results not only underscore the critical role of gut microbiota dysbiosis in PD but also identify the specific microbial species potentially contributing to PD progression.

Increasing evidence suggests that microbial metabolites mediate gut‐brain crosstalk in PD pathogenesis [4]. For example, SCFAs enhance BBB integrity and modulate neuroinflammation, thereby mitigating motor deficits in PD [4]. Besides, bile acids are reported to be altered in the plasma of PD patients, suggesting their potential role in PD [13]. Consistently, our metabolome analysis showed significantly elevated serum DCA, IDCA, and UDCA in the PD group mice, which are important secondary bile acids metabolized by gut microbiota. Specifically, conjugated primary bile acids are secreted into the intestine and first deconjugated by bile salt hydrolase (BSH) into cholic acid (CA) or chenodeoxycholic acid (CDCA). Subsequently, gut microbiota converts CA and CDCA into DCA and UDCA via 7α‐dehydroxylation, respectively. Besides, IDCA is an isomer of DCA that is thought to form via bacterial epimerization. Notably, BSH‐mediated deconjugation is key in this process, and the enzyme is widely expressed in *Bacteroides*, *Lactobacillus*, and *Bifidobacterium*, etc. [14] Among these microbes, *Bacteroides dorei* was found to elevate DCA levels in mice [15]. Consistently in our research, these bile acids were positively associated with the abundances of *B. fragilis* and *P. vulgatus*, suggesting that their BSH enzymes may increase the serum DCA, IDCA, and UDCA. Taken together, these results indicate that microbiota‐derived bile acids may contribute to the PD pathogenesis.

To elucidate the profound impact of these microbial bile acids on PD at single‐cell resolution, we conducted snRNA‐seq on the midbrain of GF mice. Specifically, snRNA‐seq found the significantly reduced endothelial cells and pericytes in the PD group mice, both critical for BBB. Studies showed that increased BBB permeability in PD may allow neurotoxic entry into the brain, thus promoting PD development [16]. Overall, these results suggest that gut microbiota may drive neurodegeneration via BBB impairments.

Furthermore, correlation analyses were performed to unravel the complex gut‐brain interactions during PD progression. We found that *B. fragilis* and *P. vulgatus* abundances were associated with serum DCA, IDCA, and UDCA, suggesting that the gut microbiota might cause PD‐like symptoms via producing these bile acids. Additionally, the serum levels of these metabolites were negatively correlated with endothelial cells and pericytes, indicating their damage to BBB structures. Consistently, a prior study reported that the gut dysbiosis induced by high‐fat diet elevates DCA levels, thus impairing the gut endothelial cells [17]. Moreover, these bile acids were significantly correlated with the behavioral performances, GI function, and neuropathological hallmarks. Taken together, these correlations suggest that increased serum bile acids may reduce the endothelial cells and pericytes of the BBB, thereby driving neurodegeneration in the SN.

As demonstrated in our correlation analyses, DCA, IDCA, and UDCA are crucial microbial metabolites potentially involved PD progression. Recently, elevated serum DCA levels have been reported in PD patients [18]. Mechanistically, DCA activates the TGR5/CCL5/CCR5 signaling pathway to promote neuroinflammation [19]. In our current research, we further validated that DCA treatment remarkably exacerbated PD‐like phenotypes in the rotenone‐induced mice and promoted dopaminergic neurodegeneration through BBB destruction.

Notably, our study shows that PD patient‐derived microbiota induces PD‐like symptoms in the GF mice. By leveraging a multi‐omics approach including midbrain snRNA‐seq, fecal metagenomics, and serum metabolomics, we originally map the gut microbiota‐driven midbrain remodeling at single‐cell resolution and identify the specific microbial metabolites mediating gut‐brain interactions in PD. Although our data suggest the involvement of gut microbiota, bile acids, and BBB integrity in PD pathogenesis, the bidirectional causal relationship between gut microbiota and PD requires further elucidation.

Our study underscores the potentially causal role of gut microbiota in PD development. Multi‐omics integration suggests that gut microbiota dysbiosis may drive the metabolic alterations that compromise BBB integrity, ultimately promoting dopaminergic neurodegeneration. Notably, microbiota‐derived bile acid DCA emerges as a critical contributor to disease progression.

## AUTHOR CONTRIBUTIONS

**Zhe Zhao**: Conceptualization; methodology; software; validation; investigation; funding acquisition; visualization; formal analysis; writing—review and editing; writing—original draft. **Jing Chen**: Methodology; investigation. **Yixuan Liu**: Methodology; formal analysis; visualization. **Shiqi Wang**: Methodology; investigation; data curation. **Danhua Zhao**: Methodology; validation; writing—review and editing. **Chaobo Bai**: Validation; investigation; writing—review and editing. **Meifang Wu**: Data curation; formal analysis; writing—review and editing. **Gaofei Hu**: Software; validation; visualization. **Yiwen Fu**: Validation; visualization; writing—review and editing. **Lu Fang**: Software; formal analysis; visualization; writing—review and editing. **Xiaoyi Liu**: Validation; Formal analysis; writing—review and editing. **Zheng Zhang**: Formal analysis; validation; writing—review and editing. **Rui Zhan**: Visualization; writing—review and editing. **Lemin Zheng**: Supervision; resources; writing—review and editing; funding acquisition; project administration. **Junliang Yuan**: Funding acquisition; project administration; supervision; resources; writing—review and editing.

## CONFLICT OF INTEREST STATEMENT

The authors declare no conflicts of interest.

## ETHICS STATEMENT

The study and all experimental procedures on human subjects were approved by the Ethics Committee of Peking University Sixth Hospital (No. 38 (2023)) and individual consent was waived. All the animal procedures were approved by the Institutional Animal Care and Use Committee of Peking University Health Science Center (No. LA2017004) in accordance with the guidelines developed by the National Institutes of Health Guidelines for the Use of Laboratory Animals.

## Supporting information


**Figure S1.** Gut microbiota transplantation from PD patients promotes PD phenotypes in the GF mice.
**Figure S2.** Single‐nucleus RNA sequencing identifies altered pathways and differentially expressed PD risk genes.
**Figure S3.** Schematic illustration of the potential mechanisms underlying the effects of gut microbiota transplantation from PD patients on the GF mice.
**Figure S4.** DCA treatment exacerbates the rotenone‐induced PD mice.

## Data Availability

The data that support the findings of this study are openly available in GSA at https://ngdc.cncb.ac.cn/bioproject/browse/PRJCA043238, reference number PRJCA043238. All the sequencing data have been deposited in GSA under submission number CRA027949 for metagenome sequencing (https://ngdc.cncb.ac.cn/gsa/browse/CRA027949), CRA028152 for snRNA‐seq raw data (https://ngdc.cncb.ac.cn/gsa/browse/CRA028152), OMIX database ID OMIX011049 for serum metabolomic data (https://ngdc.cncb.ac.cn/omix/release/OMIX011049) and OMIX011047 for snRNA‐seq data (https://ngdc.cncb.ac.cn/omix/release/OMIX011047). All data are deposited under BioProject accession number PRJCA043238 (https://ngdc.cncb.ac.cn/bioproject/browse/PRJCA043238). The data and scripts used are saved in GitHub https://github.com/ZhezhaoPKU/Trans_PD. Supplementary materials (methods, figures, graphical abstract, slides, videos, Chinese translated version, and update materials) may be found in the online DOI or iMeta Science http://www.imeta.science/.

## References

[1] Morris, Huw R. , Maria Grazia Spillantini , Carolyn M. Sue , and Caroline H. Williams‐Gray . 2024. “The Pathogenesis of Parkinson's Disease.” Lancet 403: 293–304. 10.1016/S0140-6736(23)01478-2 38245249

[2] Loh, Jian Sheng , Wen Qi Mak , Li Kar Stella Tan , Chu Xin Ng , Hong Hao Chan , Shiau Hueh Yeow , Jhi Biau Foo , et al. 2024. “Microbiota‐Gut‐Brain Axis and Its Therapeutic Applications in Neurodegenerative Diseases.” Signal Transduction and Targeted Therapy 9: 37. 10.1038/s41392-024-01743-1 38360862 PMC10869798

[3] Tan, Ai Huey , Shen Yang Lim , and Anthony E. Lang . 2022. “The Microbiome‐Gut‐Brain Axis in Parkinson Disease ‐ From Basic Research to the Clinic.” Nature Reviews Neurology 18: 476–495. 10.1038/s41582-022-00681-2 35750883

[4] Sampson, Timothy R. , Justine W. Debelius , Taren Thron , Stefan Janssen , Gauri G. Shastri , Zehra Esra Ilhan , Collin Challis , et al. 2016. “Gut Microbiota Regulate Motor Deficits and Neuroinflammation in a Model of Parkinson's Disease.” Cell 167: 1469–1480.e12. 10.1016/j.cell.2016.11.018 27912057 PMC5718049

[5] Yang, Huijia , Yaping Shao , Yiying Hu , Jin Qian , Panpan Wang , Lulu Tian , Yang Ni , et al. 2024. “Fecal Microbiota From Patients With Parkinson's Disease Intensifies Inflammation and Neurodegeneration in A53T Mice.” CNS Neuroscience & Therapeutics 30: e70003. 10.1111/cns.70003 39161161 PMC11333719

[6] Kia, Demis A. , David Zhang , Sebastian Guelfi , Claudia Manzoni , Leon Hubbard , Regina H. Reynolds , Juan Botía , et al. 2021. “Identification of Candidate Parkinson Disease Genes by Integrating Genome‐Wide Association Study, Expression, and Epigenetic Data Sets.” JAMA Neurology 78: 464–472. 10.1001/jamaneurol.2020.5257 33523105 PMC7851759

[7] Kwon, Soon‐Kyeong , Jun Chul Park , Kwang H. Kim , Jaekyung Yoon , Yejin Cho , Buhyun Lee , Jin‐Jae Lee , et al. 2022. “Human Gastric Microbiota Transplantation Recapitulates Premalignant Lesions in Germ‐Free Mice.” Gut 71: 1266–1276. 10.1136/gutjnl-2021-324489 34389621

[8] Zhao, Zhe , Jingwen Ning , Xiu‐Qi Bao , Meiyu Shang , Jingwei Ma , Gen Li , and Dan Zhang . 2021. “Fecal Microbiota Transplantation Protects Rotenone‐Induced Parkinson's Disease Mice via Suppressing Inflammation Mediated by the lipopolysaccharide‐TLR4 Signaling Pathway Through the Microbiota‐Gut‐Brain Axis.” Microbiome 9: 226. 10.1186/s40168-021-01107-9 34784980 PMC8597301

[9] Zhao, Zhe , Jing Chen , Danhua Zhao , Baoyu Chen , Qi Wang , Yuan Li , Junyi Chen , et al. 2024. “Microbial Biomarker Discovery in Parkinson's Disease Through a Network‐Based Approach.” npj Parkinson's Disease 10: 203. 10.1038/s41531-024-00802-2 PMC11513973 39461950

[10] Keshavarzian, Ali , Stefan J. Green , Phillip A. Engen , Robin M. Voigt , Ankur Naqib , Christopher B. Forsyth , Ece Mutlu , and Kathleen M. Shannon . 2015. “Colonic Bacterial Composition in Parkinson's Disease.” Movement Disorders 30: 1351–1360. 10.1002/mds.26307 26179554

[11] Hartstra, Annick V. , Valentina Schüppel , Sultan Imangaliyev , Anouk Schrantee , Andrei Prodan , Didier Collard , Evgeni Levin , et al. 2020. “Infusion of Donor Feces Affects the Gut–Brain Axis in Humans With Metabolic Syndrome.” Molecular Metabolism 42: 101076. 10.1016/j.molmet.2020.101076 32916306 PMC7536740

[12] Cerdó, Tomás , Alicia Ruiz‐Rodríguez , Inmaculada Acuña , Francisco José Torres‐Espínola , Sergio Menchén‐Márquez , Fernando Gámiz , Milagros Gallo , et al. 2023. “Infant Gut Microbiota Contributes to Cognitive Performance in Mice.” Cell Host & Microbe 31: 1974–1988.e4. 10.1016/j.chom.2023.11.004 38052208

[13] Shao, Yaping , Tianbai Li , Zheyi Liu , Xiaolin Wang , Xiaojiao Xu , Song Li , Guowang Xu , and Weidong Le . 2021. “Comprehensive Metabolic Profiling of Parkinson's Disease by Liquid Chromatography‐Mass Spectrometry.” Molecular Neurodegeneration 16: 4. 10.1186/s13024-021-00425-8 33485385 PMC7825156

[14] Song, Ziwei , Yuanyuan Cai , Xingzhen Lao , Xue Wang , Xiaoxuan Lin , Yingyun Cui , Praveen Kumar Kalavagunta , et al. 2019. “Taxonomic Profiling and Populational Patterns of Bacterial Bile Salt Hydrolase (BSH) Genes Based on Worldwide Human Gut Microbiome.” Microbiome 7: 9. 10.1186/s40168-019-0628-3 30674356 PMC6345003

[15] Sun, Xiaowei , Zhenhui Chen , Lu Yu , Weisen Zeng , Boyuan Sun , Hongying Fan , and Yang Bai . 2023. “Bacteroides Dorei BDX‐01 Alleviates DSS‐Induced Experimental Colitis in Mice by Regulating Intestinal Bile Salt Hydrolase Activity and the FXR‐NLRP3 Signaling Pathway.” Frontiers in Pharmacology 14: 1205323. 10.3389/fphar.2023.1205323 37292154 PMC10244678

[16] de Rus Jacquet, A. , M. Alpaugh , H. L. Denis , J. L. Tancredi , M. Boutin , J. Decaestecker , C. Beauparlant , et al. 2023. “The Contribution of Inflammatory Astrocytes to BBB Impairments in a Brain‐Chip Model of Parkinson's Disease.” Nature Communications 14: 3651. 10.1038/s41467-023-39038-8 PMC10282096 37339976

[17] Mouries, Juliette , Paola Brescia , Alessandra Silvestri , Ilaria Spadoni , Marcel Sorribas , Reiner Wiest , Erika Mileti , et al. 2019. “Microbiota‐Driven Gut Vascular Barrier Disruption Is a Prerequisite for Non‐Alcoholic Steatohepatitis Development.” Journal of Hepatology 71: 1216–1228. 10.1016/j.jhep.2019.08.005 31419514 PMC6880766

[18] Li, Peipei , Bryan A. Killinger , Elizabeth Ensink , Ian Beddows , Ali Yilmaz , Noah Lubben , Jared Lamp , et al. 2021. “Gut Microbiota Dysbiosis Is Associated With Elevated Bile Acids in Parkinson's Disease.” Metabolites 11: 29. 10.3390/metabo11010029 33406628 PMC7823437

[19] Zhong, Shanshan , Fangxi Liu , Rashid Giniatullin , Jukka Jolkkonen , Yong Li , Zhike Zhou , Xinyu Lin , et al. 2023. “Blockade of CCR5 Suppresses Paclitaxel‐Induced Peripheral Neuropathic Pain Caused by Increased Deoxycholic Acid.” Cell Reports 42: 113386. 10.1016/j.celrep.2023.113386 37948181

