# Supplementary material for: Multi‐omics identifies microbiota‐derived deoxycholic acid as a key mediator of blood‐brain barrier dysfunction in Parkinson's disease
Source: Imeta. 2025 Sep 17;4(5):e70076. doi: 10.1002/imt2.70076 (PMC12528001; doi:10.1002/imt2.70076)
Supplement: Supplementary file 1 — Figure S1. Gut microbiota transplantation from PD patients promotes PD phenotypes in the GF mice. Figure S2. Single‐nucleus RNA sequencing identifies altered pathways and differentially expressed PD risk genes. Figure S3. Schematic illustration of the potential mechanisms underlying the effects of gut microbiota transplantation from PD patients on the GF mice. Figure S4. DCA treatment exacerbates the rotenone‐induced PD mice. [file IMT2-4-e70076-s001.docx]

**Supplementary materials to**

**Multi-omics identifies microbiota-derived deoxycholic acid as a key mediator of blood-brain barrier dysfunction in Parkinson’s disease**

**Running title**: Multi-omics links microbial DCA to BBB dysfunction in PD

Zhe Zhao^1,2^, Jing Chen^3^, Yixuan Liu^4,5^, Shiqi Wang^4,5^, Danhua Zhao^3^, Chaobo Bai^3^, Meifang Wu^6^, Gaofei Hu^7^, Yiwen Fu^7^, Lu Fang^7^, Xiaoyi Liu^7^, Zheng Zhang^7^, Rui Zhan^8^, Lemin Zheng^7,9^*, Junliang Yuan^3^*

^1^Department of Pharmacy, Peking University Third Hospital, Beijing 100191, China

^2^Institute for Drug Evaluation, Peking University Health Science Center, Beijing 100191, China

^3^Department of Neurology, Peking University Sixth Hospital, Peking University Institute of Mental Health, NHC Key Laboratory of Mental Health (Peking University), National Clinical Research Center for Mental Disorders (Peking University Sixth Hospital), Beijing 100191, China

^4^Neuroscience Research Institute and Department of Neurobiology, School of Basic Medical Sciences, Peking University, Beijing 100083, China

^5^Key Laboratory for Neuroscience, Ministry of Education/National Health Commission, Peking University, Beijing 100083, China

^6^Department of Cardiovascular, Affiliated Hospital of Putian University, Putian 351100, China

^7^The Institute of Cardiovascular Sciences and Institute of Systems Biomedicine, School of Basic Medical Sciences, State Key Laboratory of Vascular Homeostasis and Remodeling, NHC Key Laboratory of Cardiovascular Molecular Biology and Regulatory Peptides, Beijing Key Laboratory of Cardiovascular Receptors Research, Health Science Center, Peking University, Beijing 100191, China

^8^Research Center for Cardiopulmonary Rehabilitation, University of Health and Rehabilitation Sciences Qingdao Hospital (Qingdao Municipal Hospital), School of Health and Life Sciences, University of Health and Rehabilitation Sciences, Qingdao 266113, China

^9^Beijing Tiantan Hospital, China National Clinical Research Center for Neurological Diseases, Advanced Innovation Center for Human Brain Protection, Capital Medical University, 6 Tiantan Xili, Chongwen District, Beijing 100050, China.

*Correspondence: junliangyuan@bjmu.edu.cn (Junliang Yuan); zhengl@bjmu.edu.cn (Lemin Zheng)

**METHODS**

**Human subjects and mouse colonization**

The study and all experimental procedures on human subjects were approved by Ethics Committee of Peking University Sixth Hospital (No. 38 (2023)) and individual consent was waived. In this study, human fecal samples were collected from five clinically diagnosed, treatment-naïve Parkinson’s disease (PD) patients and five matched healthy controls (HCs). These patients were diagnosed with PD at Peking University Sixth Hospital according to the Movement Disorder Society (MDS) Clinical Diagnostic Criteria for PD. Exclusion criteria for PD patient recruitment include: diagnosis of other neurological or psychiatric disorders; acute or chronic infectious diseases; history of gastrointestinal (GI) diseases; the use of antibiotics or probiotics within the past three months; chemotherapy or radiotherapy for malignancies six months prior to the sample collection. HCs were selected to closely match PD patients, like spouses of PD patients or age-matched healthy volunteers from the same geographical area with similar lifestyles. The exclusion criteria for healthy subjects were as follows: abnormal physical examinations or blood test results; digestive symptoms or any history of GI diseases; diagnosed neurodegenerative conditions; the use of probiotics, antibiotics, NSAIDs, or prescription medications six months before sample collection. The fecal samples were collected at the hospital and immediately transported to the laboratory under anaerobic conditions to preserve microbial viability. Upon arrival, the fresh samples were processed within an anaerobic workstation. Fecal samples were pooled separately for each group. A total of 500 μg of the pooled feces from each group was thoroughly dissolved in 25 mL of sterile Ringer's solution. The mixture was then filtered through a sterile 70-μm nylon mesh and centrifuged at 4,000 rpm for 15 minutes. After discarding the supernatant, the resulting pellet was resuspended in cryopreservation solution.

All the animal procedures were approved by the Institutional Animal Care and Use Committee of Peking University Health Science Center (No. LA2017004) in accordance with the guidelines developed by the National Institutes of Health Guidelines for the Use of Laboratory Animals. Male germ-free (GF) C57BL/6J mice, aging 8 weeks and weighing 20–22 g, were obtained from GemPharmatech Co., Ltd. (Nanjing, China)​. The mice were housed in ​sterile vinyl isolators​ and maintained under ​gnotobiotic conditions​ at GemPharmatech’s animal facility. All mice had free access to food and water under a strict 12-h light/dark cycle at a controlled condition (temperature 20-26°C, humidity 40–70%). The animal experimental design is illustrated in Figure. 1A. A total of 12 mice were randomly assigned to two groups: the HC group and the PD group. The fecal suspension from either PD patients or matched HCs was administered to colonize GF mice by oral gavage (200 μl per mouse) once daily for four weeks. All the mice were weighed once every two days. In addition, GI function assessments and behavioral tests were performed at week 4. At the meantime, all the mice were sacrificed for further analysis.

**PD mouse model and DCA treatment**

Male C57BL/6J mice, aged 8 weeks and weighing 20–22 g, were purchased from the Department of Experimental Animal Science, Peking University Health Science Center (Beijing, China). The mice were then acclimatized (12-h light/dark cycle) under standard conditions (temperature 22 ± 2°C, humidity 50–60%) with ad libitum access to food and water for seven days.

A total of 36 mice were randomly assigned to three groups: the Control group, the Rotenone group, and the DCA group. Rotenone (Sigma-Aldrich, St. Louis, MO, USA) dissolved in 4% carboxymethylcellulose sodium (CMC-Na; Sigma-Aldrich) with 1.25% chloroform (Beijing Chemical Works, Beijing, China) was utilized to establish a chronic PD mouse model. The mice in the Rotenone and DCA groups were orally administered with fresh rotenone solution (30 mg/kg body weight) via gavage once a day for four weeks, while the Control group mice received vehicle treatment. Simultaneously, the DCA group mice received water containing 0.2% DCA (Sigma-Aldrich) throughout the treatment period, while the mice in the Control and Rotenone groups were fed with vehicle water. GI function assessments and behavioral tests were performed at week 4. All the mice were sacrificed at week 4 for further analysis.

**Behavioral experiments**

To evaluate motor function, distinct behavioral tests were performed at hourly intervals, following protocols detailed in our prior research [1,2].

Rotarod test

Motor coordination was measured using a rotarod device. Mice were placed on the apparatus, which accelerated from 5 rpm to 40 rpm over 300 seconds, and the time until falling was recorded.

Pole test

Motor balance and agility were evaluated using a vertical pole test apparatus, comprising a smooth wooden pole (50 cm in length, 3 cm in diameter) with a wooden sphere affixed to its top. Mice were trained to descend the pole into a cage, and their performance was scored on a 0–5 scale, with 0 indicating the poorest and 5 the best performance.

Open field test

Exploratory behavior and locomotor activity were assessed in an open field arena (40 × 40 cm). Mice were placed at the center and allowed to explore for five minutes. Parameters such as the total distance traveled, time spent in central and peripheral zones, and mean speeds were recorded using the SMART 3.0 camera system (Model 64088, RWD Life Science Co., Ltd). Data were processed using SMART 3.0 Advanced Software (SMARTSUPER, Panlab, Spain). The arena was cleaned with ethanol between sessions, and tests were conducted in a quiet environment.

**GI function assessments**

Following a 2-hour fasting period, mice were placed in clean, transparent plastic cages for two hours. Fecal pellets were collected, counted, and weighed immediately to determine wet weight. After drying at 85°C for 24 hours, the dry weight was measured. The water content percentage was calculated based on the difference between wet and dry weights. To evaluate the gut motility, fecal pellets were collected over a 20-minute interval, with counts recorded every five minutes.

In addition, colon length was measured from the cecum’s distal end to the anus to assess overall colon dimensions.

**Immunohistochemical staining**

Midbrain tissues from three randomly chosen mice in each group were prepared for paraffin embedding and sectioning. Immunohistochemical (IHC) staining was conducted following the standard protocols. Initially, the tissue sections were subjected to deparaffinization and rehydration, followed by blocking with 3% bovine serum albumin to minimize nonspecific binding. The sections were then incubated overnight at 4 °C with primary antibodies targeting α-synuclein (α-syn, 1:1500, Servicebio, Wuhan, China) and tyrosine hydroxylase (TH, 1:1500, Servicebio). After extensive washing, the sections were treated with horseradish peroxidase (HRP)-conjugated secondary antibodies (goat anti-rabbit IgG or goat anti-mouse IgG, 1:200, Servicebio) for two hours at room temperature. Positive staining was visualized using 3,3'-diaminobenzidine (DAB) as the chromogenic substrate (Servicebio). Images were acquired using a Carl Zeiss microscope (Jena, Germany), and quantitative analysis of TH-positive cells or α-syn expression levels was performed using ImageJ software (version 1.52). TH-positive cells were manually counted in three randomly selected sections for each mouse, and the average number was calculated as dopaminergic neuron numbers. Optical density (OD) values were measured in three randomly selected fields for each mouse to quantify α-syn expression levels.

**Nucleus isolation and single nucleus RNA sequencing**

Midbrain tissues were collected from three randomly selected mice per group and snap-frozen in liquid nitrogen for intact nucleus isolation. Nuclei were isolated and purified using a modified protocol. Briefly, frozen tissues were homogenized in NLB buffer (250 mM Sucrose, 10 mM Tris-HCl, 3 mM MgAc2, 0.1% Triton X-100, 0.1 mM EDTA, 0.2 U/μL RNase Inhibitor) and purified using varying sucrose concentrations. The nuclei concentration was adjusted to approximately 1000 nuclei/μL for snRNA-seq.

snRNA-seq libraries were constructed using the 10 × Genomics Chromium Controller Instrument and Chromium Single Cell 3’ V3.1 Reagent Kits. Nuclei were concentrated to 1000 nuclei/μL and loaded into channels to generate single-cell Gel Bead-In-Emulsions (GEMs). Following reverse transcription, GEMs were broken, and barcoded cDNA was purified, amplified, fragmented, A-tailed, and ligated with adaptors. Index PCR was performed to amplify the final libraries. Library quantification was performed using the Qubit High Sensitivity DNA assay, and size distribution was determined using a High Sensitivity DNA chip on a Bioanalyzer 2200. Libraries were sequenced on the Novaseq6000 platform with 150 bp paired-end reads.

**SnRNA-seq data processing and bioinformatics**

Post-sequencing, the raw data underwent quality control to filter out low-quality nuclei and potential doublets. Reads were aligned to the refdata-gex-mm10-2020-A reference genome. Gene expression matrices were generated and normalized to account for sequencing depth using Cell Ranger “count” function (10 × Genomics, v.7.2.0). Then, the snRNA-seq data were processed and integrated using the Seurat package (version 4.3.0.1). The nuclei with detected feature counts between 200 and 8,000 and mitochondrial content below 10% were retained for downstream analysis. For data integration, the Harmony algorithm was employed to correct for batch effects. The integrated dataset underwent Uniform Manifold Approximation and Projection (UMAP) for dimensionality reduction using the RunUMAP function with the first 20 dimensions. Subsequently, the FindNeighbors function identified neighboring nuclei, and clustering was performed with the FindClusters function at a resolution of 0.2. Cluster-specific marker genes were identified and manually compared against reference cell-type markers from CellMarker 2.0 (<http://bio-bigdata.hrbmu.edu.cn/CellMarker>) [3]. Annotation was assigned based on the highest correspondence between the detected marker genes and known cell-type profiles. Differential gene expression (DEG) analysis between the PD and HC groups was conducted using the Seurat “FindMarkers” function. Genes with an adjusted p-value < 0.05 and an absolute average log_2_ fold change (FC) > 0.2 were considered significantly differentially expressed. Gene set enrichment analysis (GSEA) was performed to uncover the biological significance of differentially expressed genes across various cell types. The analysis utilized mouse hallmark gene sets from the MSigDB database, REACTOME pathways, and Gene Ontology Biological Process (GO BP) terms, employing the fgsea package (version 1.26.0) for statistical evaluation. We extracted the candidate PD risk genes identified by the United Kingdom Brain Expression Consortium (UKBEC) and the International Parkinson’s Disease Genomics Consortium (IPDGC) through integration of ​genome-wide association study (GWAS) data with expression datasets (Braineac, GTEx, CommonMind) using ​Coloc​ and ​transcriptome-wide association study (TWAS), focusing on the genes whose expression changes were associated with PD risk [4]. DEG analysis was then performed on these candidate genes to validate their dysregulation in the GF mice receiving fecal microbiota.

**Fecal DNA extraction and metagenomic sequencing**

At week 4, each mouse was housed in a sterile cage, and 6-8 fresh fecal pellets were collected and promptly stored at -80°C within 30 minutes. DNA extraction was performed using the MagPure Stool DNA KF Kit B (BGI-Shenzhen), following the manufacturer's protocol. Subsequently, libraries were prepared using the MGIEasy Universal DNA Library Prep Set (BGI-Shenzhen). DNA nanoballs (DNBs), which contain multiple DNA copies, were created and loaded onto nanoarrays using high-intensity DNA nanochip technology. Sequencing was carried out on the DNBSEQ-2000 platform (BGI-Shenzhen), producing PE150 reads. Raw data were processed with SOAPnuke v.2.2.1, and host-derived reads were eliminated by mapping to the host genome using SOAP2. High-quality reads were assembled de novo with MEGAHIT, excluding contigs shorter than 300 bp. Gene prediction was done using MetaGeneMark, and redundancy was minimized with CD-HIT. Gene abundance was measured using Salmon, and protein sequences were annotated by aligning them to functional databases with DIAMOND. Taxonomic classification was assigned using Kraken LCA, and abundance profiles were generated with Bracken.

**Serum metabolic profiling**

Mouse serum samples were initially thawed at 4°C and subjected to extraction using a solvent mixture of methanol, acetonitrile, and water in a 4:2:1 ratio (v/v/v), which included the internal standards for normalization. The samples were then incubated at -20°C for two hours to facilitate metabolite extraction, followed by centrifugation at 25,000 × g for 15 minutes at 4°C. The resulting supernatant was dried and subsequently reconstituted in methanol, followed by another centrifugation step to ensure purity. Quality control measures were implemented to ensure data reliability. Metabolite profiling was conducted using the Waters UPLC I-Class Plus system (Waters, USA) coupled with the QTRAP 6500 Plus mass spectrometer (SCIEX, USA). Metabolite identification was performed using Compound Discoverer 3.3 software (Thermo Fisher Scientific), with reference to databases such as bmdb, mzCloud, and ChemSpider.

For bioinformatics analysis, the raw data underwent preprocessing steps, including Probabilistic Quotient Normalization (PQN) and QC-based Robust LOESS Signal Correction (QC-RLSC), to normalize the data and correct for batch effects. Principal Component Analysis (PCA) was employed to assess sample quality and distribution. To identify significant metabolites, Partial Least Squares-Discriminant Analysis (PLS-DA) was applied, with thresholds set at a fold change (FC) > 1.5, *p*-value < 0.01, and variable importance in projection (VIP) > 1. Metabolite annotation was carried out using the Human Metabolome Database (HMDB) and the KEGG PATHWAY database, ensuring comprehensive metabolic pathway analysis.

**Midbrain bile acid quantification**

Bile acid analysis was performed using LC-ESI-MS/MS (ExionLC™ AD UHPLC coupled to QTRAP® 6500+). Midbrain samples from each group (20 mg) were homogenized and extracted with methanol/acetonitrile (2:8, v/v), followed by protein precipitation at -20°C and centrifugation (12,000 rpm, 4°C). The supernatant was analyzed on a Waters HSS T3 C18 column (100 × 2.1 mm, 1.8 µm) with a gradient of 0.01% acetic acid/5 mM ammonium acetate (A) and acetonitrile/0.01% acetic acid (B) at 0.35 mL/min. MS detection used negative ESI mode with MRM transitions, optimized for DP/CE parameters (source: 550°C, IS voltage: -4500 V). Quantitation employed internal standards and Multiquant 3.0.3 software.

**Transmission electron microscopy**

The mice were anesthetized and perfused with 0.9% saline. The brain tissues were then dissected and cut into 1 mm³ cubes. These samples were fixed in 4% paraformaldehyde at 4°C for four hours, followed by post-fixation in 1% osmium tetroxide at room temperature for two hours. The tissues were subsequently dehydrated through a graded series of ethanol, embedded in resin, and polymerized by baking at 60°C for 48 hours. Ultrathin sections (60 nm) were prepared using an ultramicrotome. The sections were examined using a transmission electron microscopy (HITACHI HT7700, Japan) to analyze the ultrastructure of the blood-brain barrier (BBB).

**Statistical analysis**

The statistical analysis was performed using GraphPad Prism 8 software or R software version 4.0.5. The data are presented as mean ± standard deviation (SD) or median ± Interquartile Range (IQR) where appropriate. For normally distributed data, Student's t-test or one-way Analysis of Variance​ (ANOVA) followed by Least Significant Difference (LSD) post-hoc tests were used for comparisons. For non-normally distributed data, Mann-Whitney U test or Kruskal-Wallis test followed by Mann-Whitney U tests was used for comparisons. Spearman correlation analysis was performed where appropriate. A P value < 0.05 was considered statistically significant.

**References**

1. Zhao, Zhe, Fangyuan Li, Jingwen Ning, Ran Peng, Junmei Shang, Hui Liu, Meiyu Shang, Xiu-Qi Bao, Dan Zhang. 2021. “Novel compound FLZ alleviates rotenone-induced PD mouse model by suppressing TLR4/MyD88/NF-κB pathway through microbiota-gut-brain axis.” *Acta Pharmaceutica Sinica. B* 11: 2859-2879. https://doi.org/10.1016/j.apsb.2021.03.020

2. Zhao, Zhe, Jingwen Ning, Xiu-Qi Bao, Meiyu Shang, Jingwei Ma, Gen Li, Dan Zhang. 2021. “Fecal microbiota transplantation protects rotenone-induced Parkinson's disease mice via suppressing inflammation mediated by the lipopolysaccharide-TLR4 signaling pathway through the microbiota-gut-brain axis.” *Microbiome* 9: 226. https://doi.org/10.1186/s40168-021-01107-9

3. Hu, Congxue, Tengyue Li, Yingqi Xu, Xinxin Zhang, Feng Li, Jing Bai, Jing Chen, et al. 2022. “CellMarker 2.0: an updated database of manually curated cell markers in human/mouse and web tools based on scRNA-seq data.” *Nucleic Acids Research* 51: D870-D6. https://doi.org/10.1093/nar/gkac947

4. Kia, Demis A., David Zhang, Sebastian Guelfi, Claudia Manzoni, Leon Hubbard, Regina H. Reynolds, Juan Botía, et al. 2021. “Identification of Candidate Parkinson Disease Genes by Integrating Genome-Wide Association Study, Expression, and Epigenetic Data Sets.” *JAMA Neurology* 78: 464-472. https://doi.org/10.1001/jamaneurol.2020.5257

**
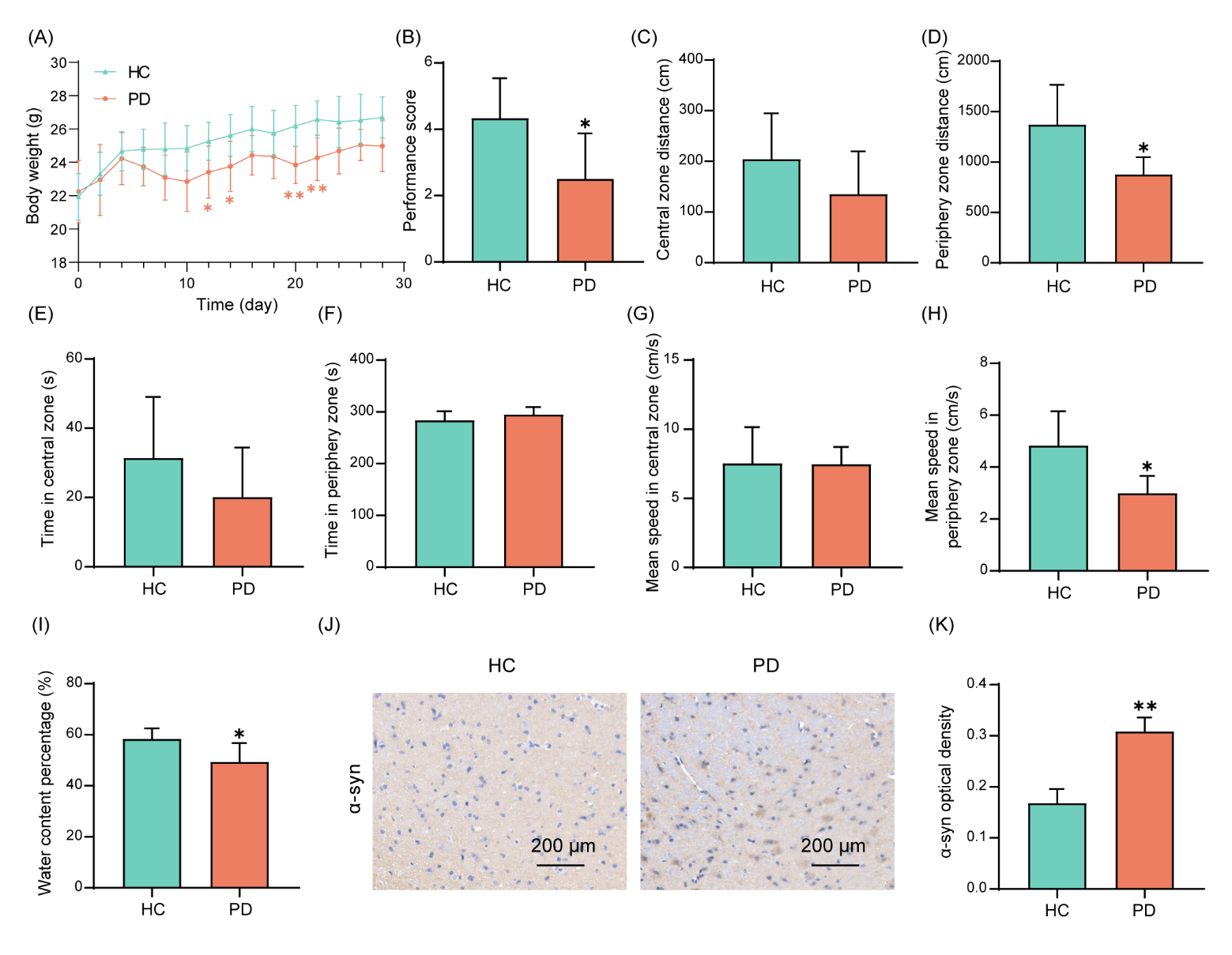
Figure S1** **Gut microbiota transplantation from PD patients promotes PD phenotypes in the GF mice.** (A) Body weights of mice during the experiment. (B) Pole test. (C) Distance traveled in the central zone. (D) Distance traveled in the periphery zone. (E) Time spent in the central zone. (F) Time spent in the periphery zone. (G) Mean speed in the central zone. (H) Mean speed in the periphery zone. (I) Water percentages of fecal pellets. (J) Representative images of IHC staining of α-syn in the SN. (K) Optical density of α-syn staining in the SN. For A-I, *n* = 6 in each group. For K, *n* = 3 in each group. Data are presented as mean ± SD. Statistics calculated by Student's t-test (A and C-K) or Mann-Whitney U tests (B). * *p* < 0.05, ** *p* < 0.01 versus the HC group. α-syn, α-synuclein; HC, healthy control; PD, Parkinson’s disease.


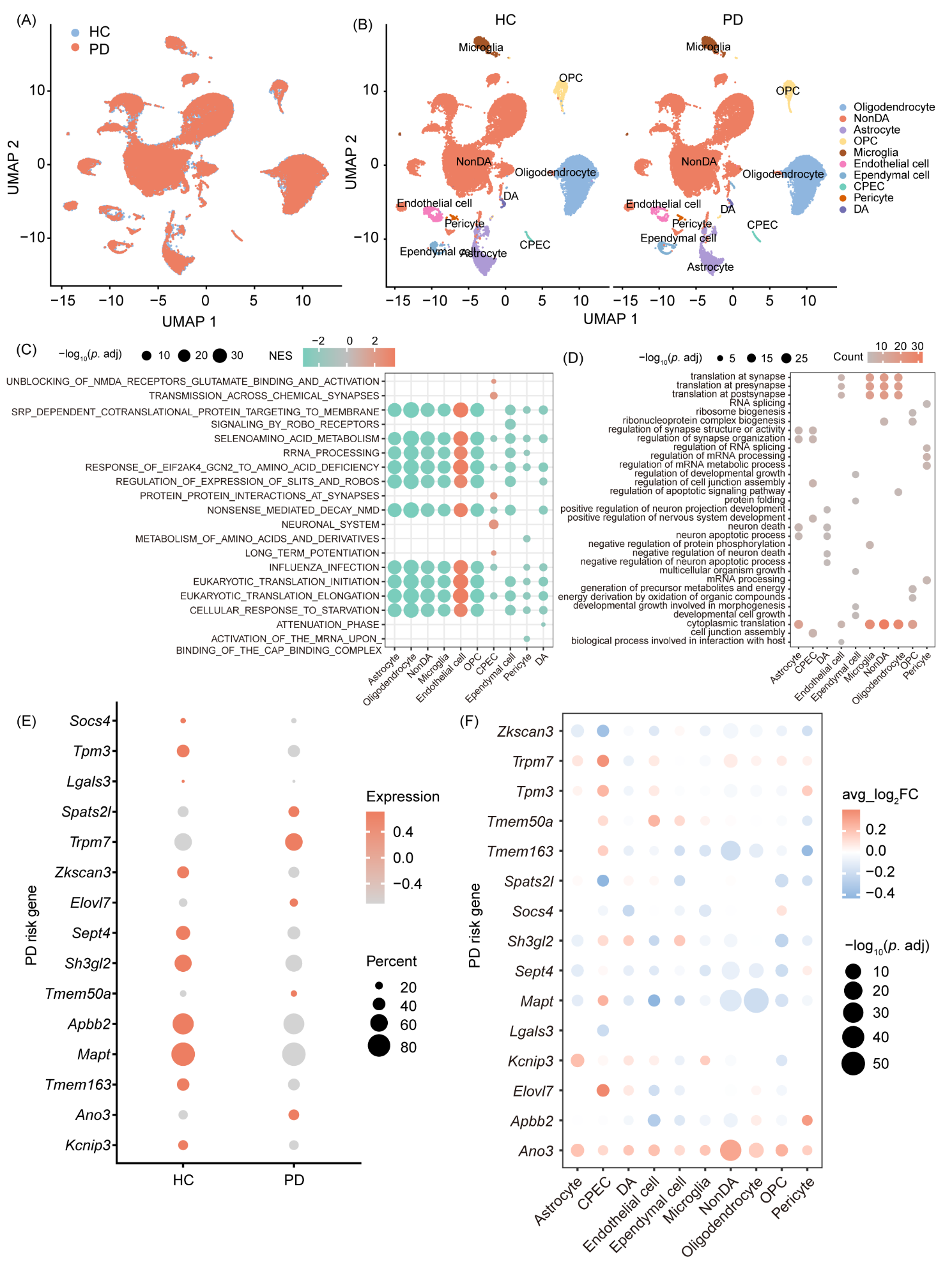
Figure S2 Single-nucleus RNA sequencing identifies altered pathways and differentially expressed PD risk genes. (A) UMAP illustration of cells colored by groups. (B) UMAP illustration of cells colored by cell types in separate groups. (C) GSEA analysis of REACTOME pathways in different cell types. (D) GSEA analysis of GO BP pathways in different cell types. (E) Dot plot showing differentially expressed PD risk genes in the whole midbrain. (F) Dot plot showing differentially expressed PD risk genes across various cell types. CPEC, choroid plexus epithelial cells; DA, dopaminergic; FC, fold change; HC, healthy control; NES Normalized Enrichment Score; NonDA, non-dopaminergic; OPC, oligodendrocyte precursor cells; *p*.adj, adjusted *p* value; PD, Parkinson’s disease; UMAP, Uniform Manifold Approximation and Projection.


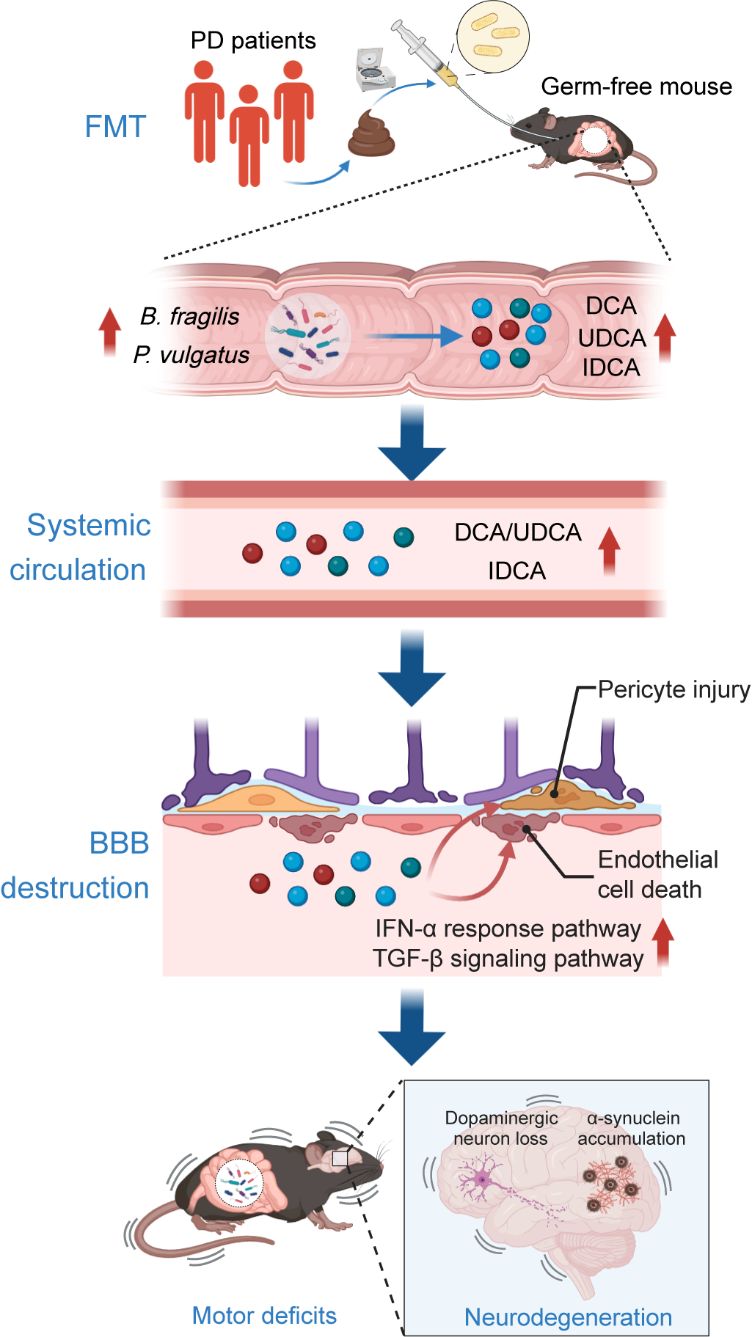
Figure S3 Schematic illustration of the potential mechanisms underlying the effects of gut microbiota transplantation from PD patients on the GF mice. The gut microbiota derived from PD patients, enriched in *B. fragilis* and *P. vulgatus*, may promote the elevations of DCA, IDCA, or UDCA levels in the systemic circulation. The increased serum bile acids in turn contributes to the endothelial cell death and pericyte injury possibly through activating interferon alpha response and TGF-β signaling pathways at the BBB in the midbrain, ultimately leading to the neurodegeneration and motor deficits. Created with BioRender.com. BBB, blood-brain barrier; *B. fragilis*, *Bacteroides fragilis*; DCA, deoxycholic acid; FMT, fecal microbiota transplantation; IDCA, iso-deoxycholic acid; *P. vulgatus*, *Phocaeicola vulgatus*; UDCA, ursodeoxycholic acid.


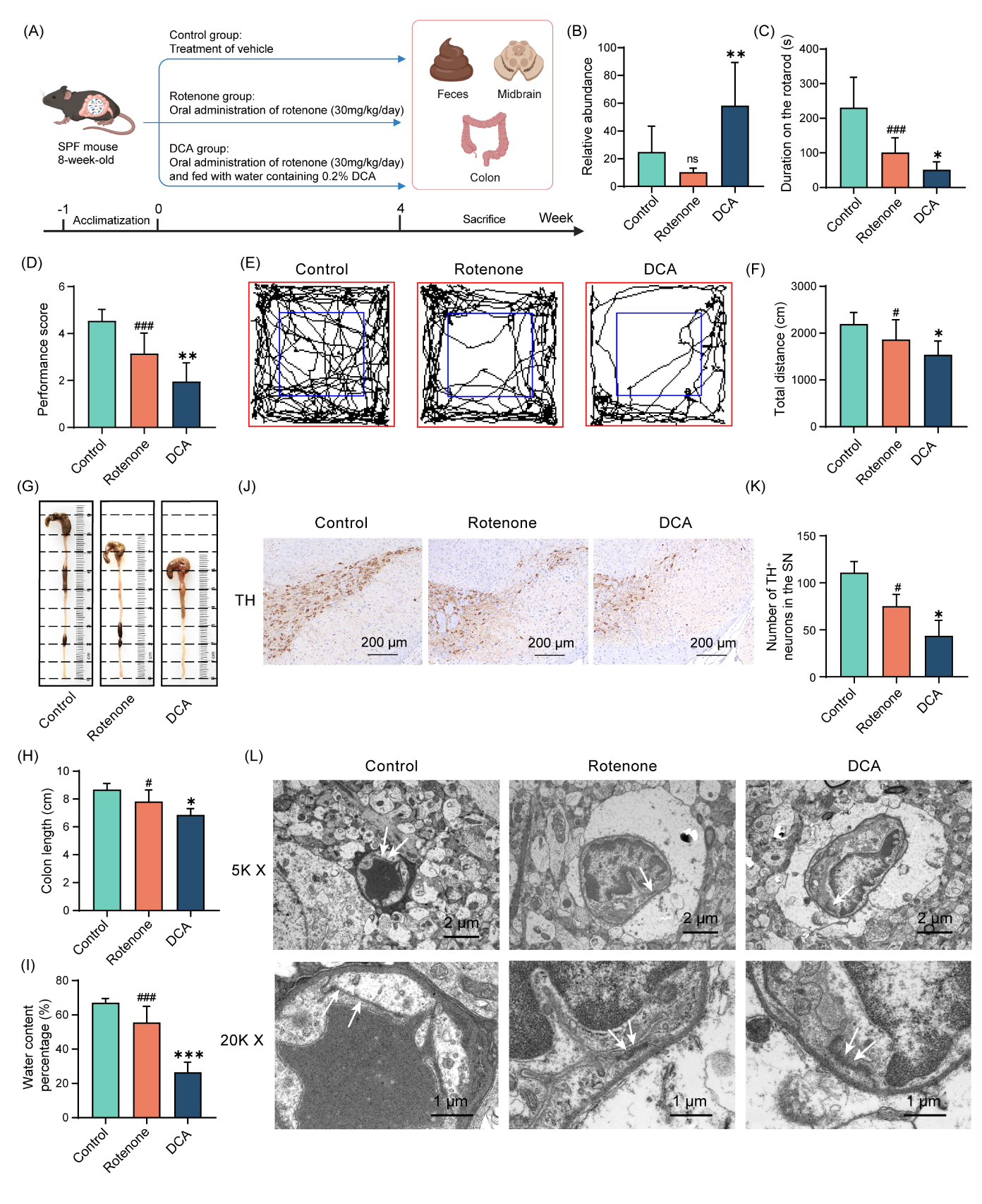
Figure S4 DCA treatment exacerbates the rotenone-induced PD mice. (A) Flow chart of experimental design. Created with BioRender.com. (B) Quantification of DCA in the midbrain. (C) Rotarod test. (D) Pole test. (E) Representative trajectory map of open field test. (F) Total distance travelled in the whole open field. (G) Representative images of colon. (H) Colon lengths. (I) Water percentages of fecal pellets. (J) Representative images of IHC staining of TH^+^ neurons in the SN. (K) Numbers of TH^+^ neurons in the SN. (L) Representative electron micrographs of the tight junction structures of BBB in the SN. For B, *n* = 6 in each group. For C-F and I, *n* = 12 in each group. For H, *n* = 5 in each group. For K, *n* = 3 in each group. Statistics calculated by one-way ANOVA followed by LSD tests (B-C, F-K) or Kruskal-Wallis test followed by Mann-Whitney U tests (D). # *p* < 0.05, ### *p* < 0.001 versus the Control group; * *p* < 0.05, ** *p* < 0.01, *** *p* < 0.001 versus the Rotenone group. DCA, deoxycholic acid; SN, substantia nigra; TH, tyrosine hydroxylase.
